# P-1567. Urinary Tract Infections Broadly Impact Quality of Life Among People with Neurogenic Bladder

**DOI:** 10.1093/ofid/ofae631.1734

**Published:** 2025-01-29

**Authors:** Margaret A Fitzpatrick, Marissa Wirth, Pooja Solanki, Katie J Suda, Stephen Burns, Frances Weaver, Eileen Collins, Nasia Safdar, Charlesnika T Evans

**Affiliations:** Rocky Mountain Regional VA Medical Center, Aurora, Colorado; Edward Hines Jr. VA Hospital, Hines, Illinois; Edward Hines, Jr. VA Hospital, Hines, Illinois; Center for Health Equity Research and Promotion and University of Pittsburgh, Pittsburgh, Pennsylvania; VA Puget Sound Health Care System, Seattle, Washington; Center of Innovation for Complex Chronic Healthcare, Hines, Illinois; University of Illinois Chicago, Chicago, Illinois; William S. Middleton VA Hospital, Madison, Wisconsin; Northwestern University and VA, Hines, Illinois

## Abstract

**Background:**

Although recurrent urinary tract infections (UTI) are common complications in people with neurogenic bladder (NB), limited data exist on the specific ways UTIs impact quality of life (QoL) in this population. These data could help tailor patient-centered approaches to improving UTI care in people with NB.

UTI Impacts on Feelings and Emotional Well-being

**Figure:**
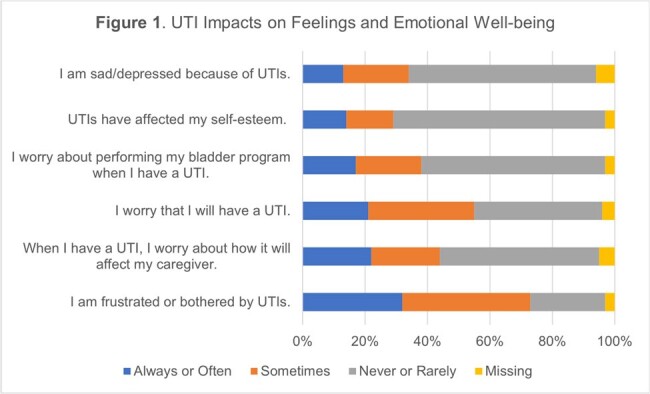

**Methods:**

We mailed surveys to 289 Veterans with NB due to spinal cord injury/disorder (SCI/D), multiple sclerosis, or Parkinson’s Disease who had at least one in-person encounter associated with a UTI diagnosis at four Veterans Affairs Medical Centers between May 2022-May 2023. The survey was adapted from existing instruments and previously collected qualitative data. An expert panel refined the survey using a Delphi process, followed by cognitive interviews with Veterans with NB (n=8). The QoL section contained 15 survey items with 5-point Likert scale response options (higher values = greater QoL impact) which assessed how UTIs affect feelings, emotional well-being, daily activities, sex life, and social participation. To account for missing and ‘not applicable’ responses, scaled QoL scores were calculated by multiplying total survey score by the ratio of 15 / the number of items answered. Descriptive statistics summarized respondent characteristics and QoL scores.

UTI Impacts on Activities

**Figure:**
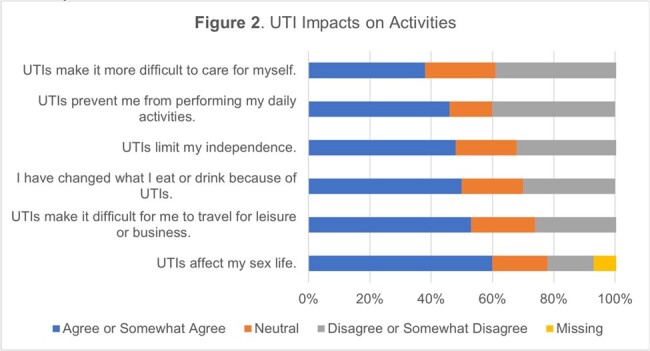

**Results:**

Respondents (n=71) were older [median age (range) = 72 (36-88)], male (92%), and white (79%). Most had SCI/D (77%) and used urinary catheters (77%). 85% of respondents answered at least 13 items. Other than feeling often or always frustrated or bothered by UTIs (n=22; 31%), most respondents did not report that UTIs frequently impact feelings or emotional well-being (Figure 1). In contrast, UTIs had a strong impact on activities (Figure 2), with many respondents agreeing their UTIs have impacted diet (n=35; 50%), travel (n=37; 53%), and sex life (n=24/40 responses; 60%). Mean [standard deviation (SD)] scaled total QoL score was 40.8 (15.3) out of a possible maximum score of 75, with a mean (SD) per item score of 2.7 (1.0) out of 5.

**Conclusion:**

People with NB experience substantial QoL impacts from UTIs, particularly on their daily and social activities. Patient-centered interventions focused on reducing negative impacts on QoL from UTIs are urgently needed for this population.

**Disclosures:**

**All Authors**: No reported disclosures

